# Strict Host-Symbiont Cospeciation and Reductive Genome Evolution in Insect Gut Bacteria

**DOI:** 10.1371/journal.pbio.0040337

**Published:** 2006-10-10

**Authors:** Takahiro Hosokawa, Yoshitomo Kikuchi, Naruo Nikoh, Masakazu Shimada, Takema Fukatsu

**Affiliations:** 1 Institute for Biological Resources and Functions, National Institute of Advanced Industrial Science and Technology (AIST), Tsukuba, Japan; 2 Department of Systems Sciences, University of Tokyo, Meguro, Tokyo, Japan; 3 Department of Molecular and Cell Biology, University of Connecticut, Storrs, Connecticut, United States of America; 4 Division of Natural Sciences, University of the Air, Chiba, Japan; University of California Davis, United States of America

## Abstract

Host-symbiont cospeciation and reductive genome evolution have been identified in obligate endocellular insect symbionts, but no such example has been identified from extracellular ones. Here we first report such a case in stinkbugs of the family Plataspidae, wherein a specific gut bacterium is vertically transmitted via “symbiont capsule.” In all of the plataspid species, females produced symbiont capsules upon oviposition and their gut exhibited specialized traits for capsule production. Phylogenetic analysis showed that the plataspid symbionts constituted a distinct group in the γ-*Proteobacteria,* whose sister group was the aphid obligate endocellular symbionts *Buchnera.* Removal of the symbionts resulted in retarded growth, mortality, and sterility of the insects. The host phylogeny perfectly agreed with the symbiont phylogeny, indicating strict host-symbiont cospeciation despite the extracellular association. The symbionts exhibited AT-biased nucleotide composition, accelerated molecular evolution, and reduced genome size, as has been observed in obligate endocellular insect symbionts. These findings suggest that not the endocellular conditions themselves but the population genetic attributes of the vertically transmitted symbionts are probably responsible for the peculiar genetic traits of these insect symbionts. We proposed the designation “*Candidatus* Ishikawaella capsulata” for the plataspid symbionts. The plataspid stinkbugs, wherein the host-symbiont associations can be easily manipulated, provide a novel system that enables experimental approaches to previously untouched aspects of the insect-microbe mutualism. Furthermore, comparative analyses of the sister groups, the endocellular *Buchnera* and the extracellular *Ishikawaella,* would lead to insights into how the different symbiotic lifestyles have affected their genomic evolution.

## Abstract 2

Evidence of host-symbiont cospeciation in an insect gut symbiont suggests that long-term vertical transmission and population structure are central forces driving the genomic changes characteristic of insect nutritional symbionts.

## Introduction

Symbiotic microorganisms are universally found in the gut, body cavity, or cells of a wide array of insects. Some obligate symbionts are of a mutualistic nature and contribute to the fitness of their hosts, while other facultative symbionts are rather parasitic and tend to cause negative effects on their hosts [1,2]. In particular, the most intimate mutualistic associations are found in obligate endocellular symbionts like *Buchnera* in aphids and *Wigglesworthia* in tsetse flies. In these insects, the symbiotic bacteria are housed in the cytoplasm of large specialized cells called bacteriocytes or mycetocytes. In the body of the insects, these cells aggregate into a large symbiotic organ called bacteriome or mycetome [3], where the inhabiting symbionts play their physiological roles such as provisioning of essential nutrients for the host insects [1,4–6]. The symbionts are vertically transmitted to the next generation in the maternal body at early stages of oogenesis or embryogenesis, where the symbiont transmission is integrated into the intricate developmental process of the host insects [3,7]. In these cases, neither the host nor the symbiont can survive without their partner, constituting an almost inseparable biological entity.

As such, the host-symbiont integrity is also corroborated by phylogenetic lines of evidence. In endocellular bacterial lineages such as *Buchnera* in aphids [8], *Carsonella* in psyllids [9], *Portiera* in whiteflies [10], *Tremblaya* in mealybugs [11], *Baumannia* in sharpshooters [12], *Blochmannia* in carpenter ants [13], *Wigglesworthia* in tsetse flies [14], *Nardonella* in weevils [15], and others, the host phylogeny generally mirrors the symbiont phylogeny, suggesting host-symbiont cospeciation over evolutionary time. It is widely thought that strict vertical transmission is the primary basis of such cocladogenesis between the symbiotic partners [8–15].

In most of these endocellular bacterial lineages in common, remarkable evolutionary patterns, including AT-biased nucleotide composition, accelerated molecular evolution, and reduced genome size have been detected in comparison with their free-living relatives [16,17]. The evolutionary patterns suggest that the endocellular lifestyle of the obligate insect symbionts might have strongly influenced their genome evolution, whose underlying mechanisms are of great interest [17,18].

In diverse insects and other organisms, symbiotic microorganisms are harbored in their gut cavity. While most of the gut microbes are commensals or parasites, some of them are known to play substantial biological roles for their hosts. For example, the gut microbial community is needed for cellulose digestion in termites [19], the gut symbiotic fungus is involved in sterol biosynthesis in anobiid beetles [20], and the gut symbiotic bacterium is essential for nymphal growth in stinkbugs [21,22]. Certainly these gut symbionts are vertically transmitted and important for their host insects, but such extracellular associations are thought to be evolutionarily more casual than the endocellular associations, on the grounds that the symbionts are not isolated in the body cavity and vulnerable to invasion and replacement by foreign microbes [3]. In termites and alydid stinkbugs, phylogenetic relationships of the gut symbionts did not mirror those of their host insects, indicating promiscuous host-symbiont associations over evolutionary time [23–25].

The stinkbugs of the family Plataspidae harbor a bacterial symbiont in the posterior midgut and are known for their unique mechanism for vertical transmission called “symbiont capsule” [22,26–28]. When deprived of the symbiont, the host insects exhibit retarded nymphal growth [22,27]. When the female insects lay eggs on their host plant, small brownish particles are always deposited under the egg mass. The particles encase a copious amount of the symbiont inside, and hatchlings from the eggs orally acquire the symbiont from the capsule (Video S1) [22,26–28].

In this study, we identified an unexpectedly intimate evolutionary association between the plataspid stinkbugs and their gut symbiotic bacteria: the symbiont phylogeny perfectly agreed with the host phylogeny, and the symbionts consistently exhibited AT-biased nucleotide composition, accelerated molecular evolution, and reduced genome size. This study first reports the strict host-symbiont cospeciation and reductive genome evolution in a group of gut bacteria and provides general and novel insights into how endocellular and extracellular symbiotic lifestyles have influenced the genome evolution of insect-associated microbes.

## Results/Discussion

### Specialized Traits for Capsule Production in Posterior Midgut of Female Insects

**Figure 1:**
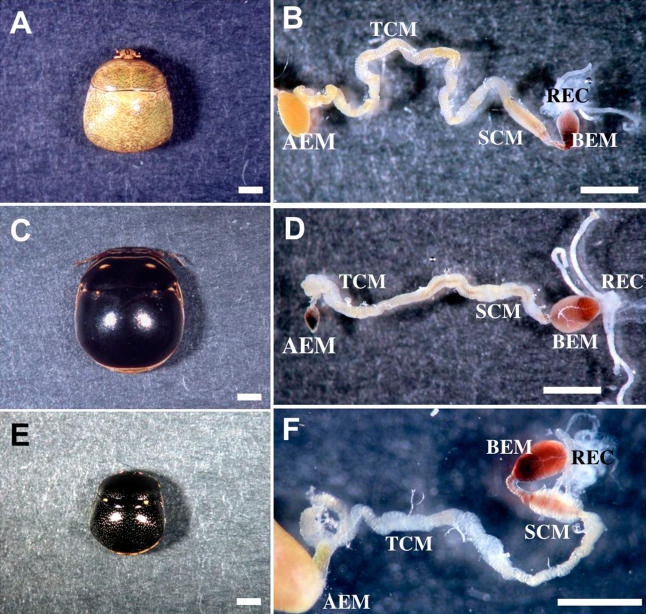
Adult Females of the Plataspid Stinkbugs and Their Posterior Midgut (A and B) *M. cribraria;* (C and D) *B. subaeneus;* (E and F) *C. parvipictum.* Bars, 1 mm. AEM, anterior enlarged midgut section; BEM, brownish enlarged midgut end section; REC, rectum; SCM, swollen crypt-bearing midgut section [28].

It was described that in Coptosoma scutellatum and *Megacopta punctatissima* the posterior midgut of female adults is differentiated into distinct sections specialized for capsule production, including the thin crypt-bearing midgut (TCM) for harboring the symbionts, the swollen crypt-bearing midgut for secretion of the matrix embedding the symbionts in the capsules, and the brownish enlarged midgut for production of the envelope encasing the capsule contents [22,26–28]. In Megacopta cribraria (Figure 1A), Brachyplatys subaeneus (Figure 1C), and Coptosoma parvipictum (Figure 1E), the same organization of the posterior midgut was identified in adult females (Figure 1B, 1D, and 1F). As for *Brachyplatys vahlii, Coptosoma sphaerula,* and *Coptosoma japonicum,* the posterior midgut of adult females also exhibited the same anatomical traits (unpublished data). In all the species, the posterior midgut of male adults substantially consisted of the TCM only, lacking the swollen crypt-bearing midgut and the brownish enlarged midgut (unpublished data). These results indicated that the traits specialized for capsule production in the posterior midgut of female insects are highly conserved among the diverse plataspid stinkbugs.

### Capsule Production by Female Insects

It was reported that in C. scutellatum and *M. punctatissima,* small particles dark in color (so-called symbiont capsules) are deposited on the underside of egg masses [22,26–28]. In *M. cribraria, B. subaeneus,* and *C. parvipictum,* all the field-collected and laboratory-born egg masses contained symbiont capsules (Figure S1). In the field-collected egg masses of *B. vahlii, C. sphaerula,* and *C. japonicum,* the symbiont capsules were always found (unpublished data). Thus, the capsule production by ovipositing female insects is a conserved trait among the diverse plataspid stinkbugs.

### Bacterial Genotyping in Posterior Midgut and Capsule

We dissected the TCM section of the posterior midgut from female adults of the stinkbugs and extracted DNA from the samples individually. Bacterial *16S rRNA* gene was amplified by PCR from each of the samples, the PCR products were cloned, and more than ten clones for each of the samples were subjected to restriction fragment length polymorphism genotyping. The restriction fragment length polymorphism patterns were identical among the clones from the same sample, were identical among the samples of the same species, and were not always identical between different species (unpublished data). We also analyzed the DNA samples extracted from isolated capsules and obtained the same results (unpublished data). These results strongly suggest that a single and specific bacterium is associated with each of the plataspid stinkbugs.

### Phylogenetic Placement of the Symbiotic Bacteria

**Figure 2:**
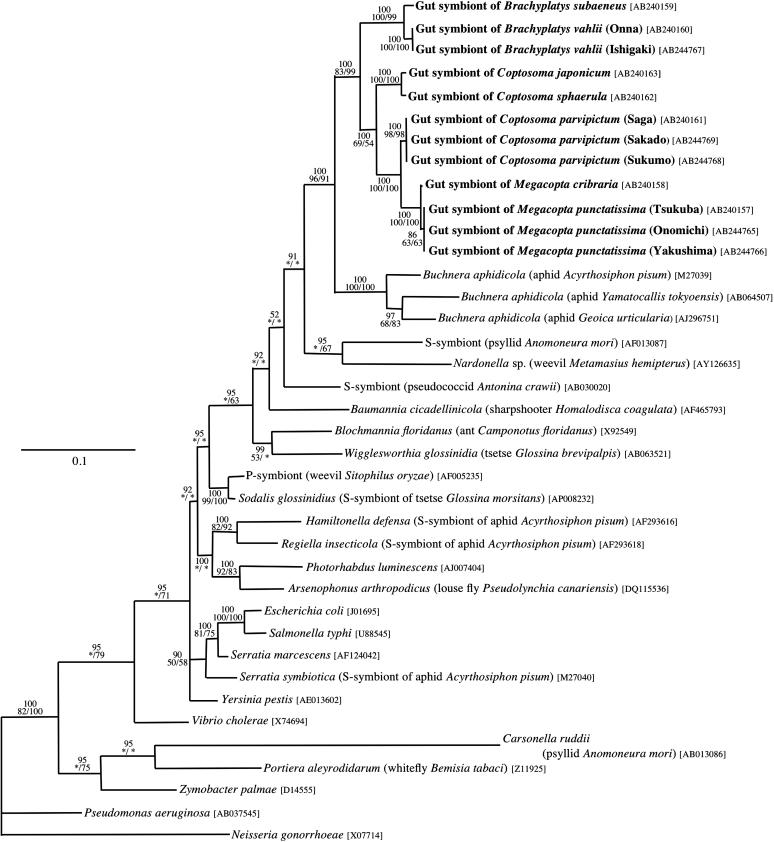
Phylogenetic Placement of the Symbiotic Bacteria from the Plataspid Stinkbugs in the γ-Subclass of the *Proteobacteria* on the Basis of *16S rRNA* Gene Sequences A total of 1,213 aligned nucleotide sites were subjected to the analysis. A Bayesian tree is shown, while MP tree and ML tree exhibited substantially the same topologies. On each of the nodes, posterior probabilities in the Bayesian analysis are shown above, and bootstrap probabilities (MP analysis/ML analysis) are shown below. Asterisks indicate bootstrap values lower than 50%. In brackets are sequence accession numbers. In parentheses are collection localities for the stinkbug symbionts and host insect names for the other symbionts, respectively.

Three or more clones from each of the midgut DNA samples were sequenced. The sequences from each of the samples were identical. The bacterial *16S rRNA* gene sequences were subjected to molecular phylogenetic analysis (Figure 2). The sequences formed a monophyletic group with high statistical supports: 100% in Bayesian; 83% in maximum parsimony (MP); 99% in maximum likelihood (ML), placed in the γ3-subclass of the *Proteobacteria.* The sister group of the plataspid symbionts was identified to be *Buchnera aphidicola,* the obligate endocellular symbionts of aphids. Phylogenetic affinity between these symbiont clades received good statistical supports: 100% in Bayesian; 96% in MP; 91% in ML. No other bacterial *16S rRNA* gene sequences in the DNA databases were placed in these clades. These results indicated that the capsule-transmitted gut symbionts of the plataspid stinkbugs comprise a distinct and coherent bacterial group in the γ-*Proteobacteria.*

### Implication for the Evolutionary Origin of Obligate Endocellular Bacterial Symbionts

It has been advocated that intimate insect-microbe endocellular associations might have evolved from more casual associations like insect gut microflora (see [3]). Recent molecular phylogenetic studies certainly revealed that many, if not all, of the obligate endocellular symbionts of diverse insects commonly belong to the same bacterial group, the γ3-subclass of the *Proteobacteria* or formerly the Enterobacteriaceae [12,13,29,30], which embraces a number of major gut bacteria of vertebrates, insects, and other invertebrates [31,32] (see Figure 2). The phylogenetic affinity between the gut symbiotic bacteria of plataspid stinkbugs and the obligate endocellular symbiotic bacteria *Buchnera* of aphids (Figure 2) is intriguing in this context. Conceivably, though speculative, the common ancestor of these symbiont clades might be a gut bacterium of ancestral hemipteran insects.

### Are the Symbiotic Bacteria Essential for Plataspid Stinkbugs?

In aphids, the endocellular symbiont *Buchnera* plays essential roles for the host insect, such as provision of essential amino acids and other nutrients that are scarce in the plant sap diet [1,4,5]. When experimentally deprived of *Buchnera,* the aphids suffer retarded growth, high mortality, and sterility [4,33]. The phylogenetic affinity to *Buchnera* led us to the idea that the plataspid symbionts might have similar biological roles for their host insect. Actually, in previous studies on C. scutellatum and *M. punctatissima,* deprivation of the symbiont capsules resulted in retarded nymphal growth [22,27]. Then, the question is whether or not the gut symbiotic bacteria are essential for survival and reproduction of the plataspid stinkbugs in general, as *Buchnera* for aphids.

### Removing Capsules from Egg Masses for Production of Symbiont-Free Insects

We divided each of the egg masses of the plataspid stinkbugs into two portions. One of the halves was left untreated, the other of the halves was deprived of all capsules, and newborn nymphs from these experimental egg masses were subjected to PCR detection of the symbiont after 1 d of hatching. Almost all the nymphs from the control egg masses with capsules possessed the symbiont, whereas most of the nymphs from the treated egg masses without capsules failed to acquire the symbiont (Table S1). In some of the nymphs from the treated egg masses, a faint band was detected with 35 cycles of PCR reaction, although the signal was not seen with 30 cycles (Table S1; Figure S2). Probably these nymphs managed to acquire a very small amount of the symbiont by probing the debris of the capsules adhering on the egg surface. In this way, we successfully obtained sibling populations of symbiotic and aposymbiotic insects in the four plataspid representatives, *M. punctatissima, M. cribraria, B. subaeneus,* and *C. parvipictum.*

### Effect of Symbiont Elimination on Adult Emergence Rate

**Figure 3:**
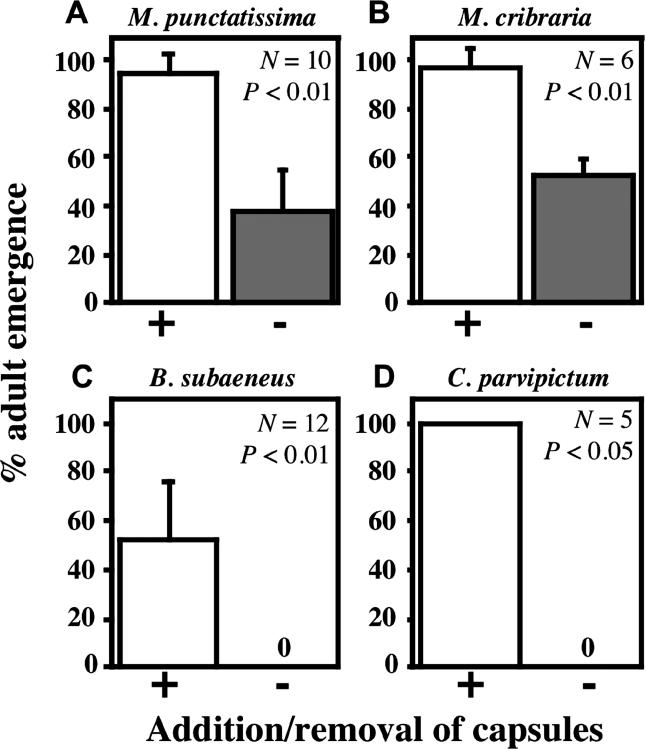
Effects of Symbiont Elimination on the Adult Emergence Rate of the Plataspid Stinkbugs (A) *M. punctatissima;* (B) *M. cribraria;* (C) *B. subaeneus;* (D) *C. parvipictum.* Means and standard deviations are shown. Open columns, with capsules; filled columns, without capsules. Sample sizes and *p* values of Wilcoxon test are indicated.

Figure 3 shows the adult emergence rate from the control egg masses and the treated egg masses. In all of the four species, the adult emergence rate without the symbiont capsules was drastically reduced in comparison with that with the symbiont capsules. In M. punctatissima and *M. cribraria,* about 50% of the aposymbiotic nymphs died during developmental course (Figure 3A and 3B). In B. subaeneus and *C. parvipictum,* all of the aposymbiotic nymphs died before adult emergence (Figure 3C and 3D).

### Effect of Symbiont Elimination on Developmental Time

Table 1 shows the developmental time of the adult insects of M. punctatissima and M. cribraria that emerged from the experimental egg masses. In both of the species and irrespective of the sexes, the developmental time of the aposymbiotic insects was significantly prolonged in comparison with that of the symbiotic insects.

**Table 1:**
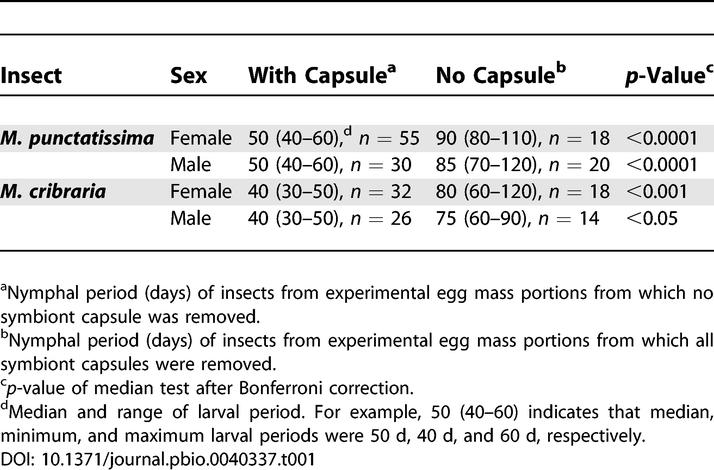
Developmental Time of Stinkbug Nymphs from Experimental Egg Masses with and without Capsules

### Effect of Symbiont Elimination on Adult Body Size and Phenotype

**Figure 4:**
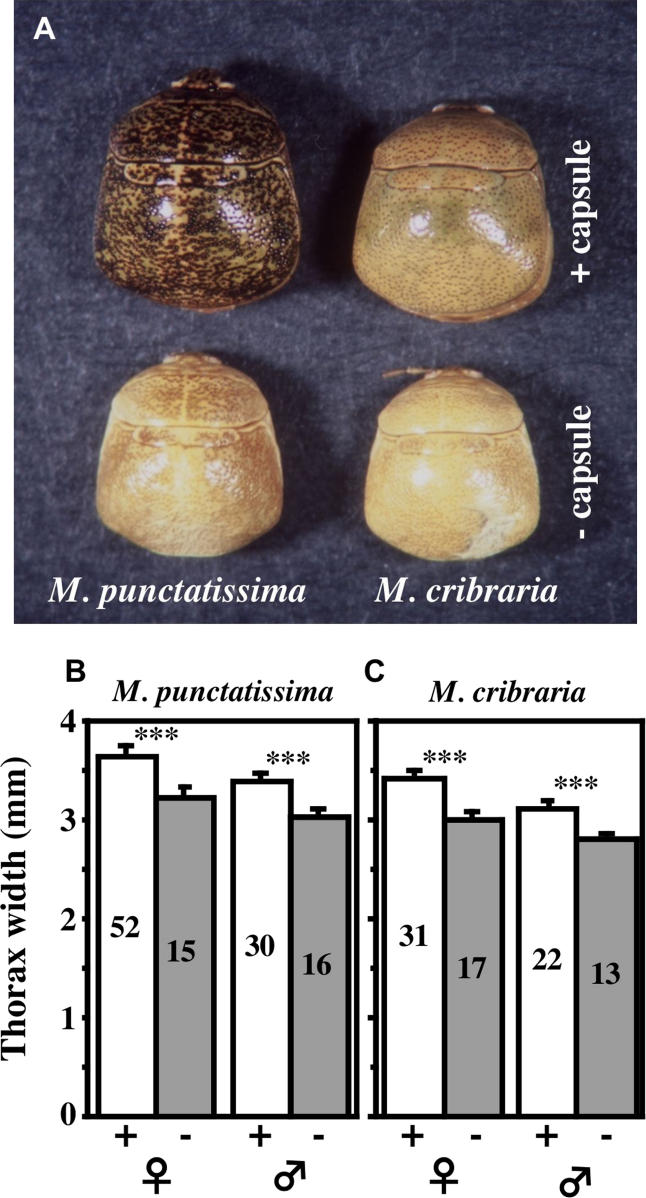
Effects of Symbiont Elimination on Adult Body Size and Phenotype of the Plataspid Stinkbugs (A) Adult females of M. punctatissima (left) and M. cribraria (right) emerged from the control egg masses with capsules (top) and those from the treated egg masses without capsules (bottom). (B) Thorax width of M. punctatissima and (C) thorax width of *M. cribraria.* Means and standard deviations are shown. Open columns, with capsules; filled columns, without capsules. Sample sizes are labeled on the columns. Asterisks indicate statistically significant differences (median test; *** *p* < 0.001 after Bonferroni correction).

Figure 4 shows the body size of the adult insects of M. punctatissima and M. cribraria that emerged from the experimental egg masses. In both of the species and irrespective of the sexes, the adult insects from the control egg masses with capsules were normal in color and larger in size, while the adult insects from the treated egg masses without capsules were pale in color and smaller in size (Figure 4A). Semi-quantitative PCR analyses revealed that some of the latter insects were completely free of the symbiont, whereas the other insects were certainly infected but their symbiont titers were disproportionally smaller than those of the normal insects (unpublished data). Morphometric analyses showed that the thorax width of the aposymbiotic insects was significantly smaller than that of the normal insects (Figure 4B and 4C). These aposymbiotic adults neither copulated nor reproduced (unpublished data).

### The Symbiotic Bacteria Are Essential for Normal Development and Reproduction of the Plataspid Stinkbugs

From these results, it was concluded that the capsule-transmitted gut symbiotic bacteria are essential for normal development and reproduction of the plataspid stinkbugs. Although only four species from three genera were experimentally inspected in this study, on account of the conserved symbiotic organization (Figure 1) [22,26,28] and the phylogenetic integrity of the symbionts (Figure 2), we expect that the role of the symbiont is conserved across other plataspid stinkbugs in general. The gut symbiotic bacteria can be regarded as obligate mutualistic associates for the plataspid stinkbugs, as the endocellular symbiotic bacteria *Buchnera* for the host aphids.

### Biological Roles of the Symbiotic Bacteria for the Host Insects?

The mechanism whereby the gut symbiotic bacteria support the growth and reproduction of the plataspid stinkbugs is intriguing but totally unknown. Probably the symbiont provides the host with nutritional supplements, such as essential amino acids and vitamins, as has been reported for other plant-sucking insects [1,4,5]. To understand the physiological basis of the symbiotic association, a nutritionally defined artificial diet should be developed for the plataspid stinkbugs.

### Posterior Midgut as Specialized Symbiotic Organ

Anatomically, plataspid stinkbugs are very unique in that their alimentary tract is completely disconnected in the midway. In newborn nymphs, their gut is normally organized, which allows the ingested symbiont to colonize the midgut. In the developmental course, however, the midgut is constricted and separated into anterior and posterior parts. In adult insects, the anterior midgut is free of the symbiont, connected to the posterior midgut only with a delicate membranous thread without cavity [22,26]. Judging from the peculiar anatomy, the plant sap ingested by the insect is completely absorbed in the anterior midgut, the waste is excreted through the Malpighian tubules into the hindgut, and there is no food flow through the posterior midgut. The posterior midgut is transformed into a voluminous organ for harboring a huge amount of the symbiont in the cavity (Figure 1) [22,26,28]. The posterior midgut of plataspid stinkbugs can be regarded as “pseudo-bacteriome,” in that the obligate symbiotic bacteria are harbored not in the cytoplasm but in the extracellular space.

### Comparison of the Symbiont Phylogeny with the Host Phylogeny

**Figure 5:**
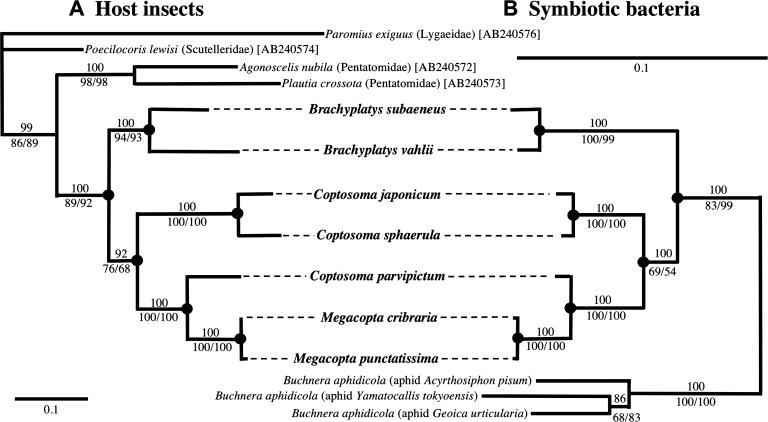
Phylogenetic Congruence between the Plataspid Stinkbugs and Their Symbiotic Bacteria (A) A Bayesian phylogeny of the host insects on the basis of mitochondrial *16S rRNA* gene sequences (847 aligned nucleotide sites). In addition to the plataspid stinkbugs, four pentatomomorphan species were examined as out-group taxa. In parentheses are taxonomic affiliations and in brackets are sequence accession numbers. (B) A Bayesian phylogeny of the plataspid symbionts on the basis of the same data of Figure 2 (*16S rRNA* gene, 1,213 aligned nucleotide sites). Dots indicate codivergence events inferred by the jungles algorithm [34]. Posterior probabilities in the Bayesian analysis and bootstrap values in the MP/ML analyses are presented as shown in Figure 2.

Figure 5 shows the comparison between the host phylogeny and the symbiont phylogeny of the plataspid stinkbugs. The molecular phylogeny based on mitochondrial *16S rRNA* gene sequences reflected the systematics of the plataspid stinkbugs: Megacopta spp. and Brachyplatys spp. formed monophyletic groups, respectively, while Coptosoma spp. were paraphyletic (Figure 5A). Strikingly, the phylogenetic relationship of the host insects perfectly agreed with the phylogenetic relationship of their symbiotic bacteria (Figure 5B). On account of a total of 10,395 possible rooted tree topologies for seven taxa, the chance that the symbiont tree will exactly match the host tree is expected to be less than 0.0001. The congruence was so complete that the jungles algorism [34] identified only one reconstruction of the coevolutionary history with six codivergence events (see Figure 5). A randomization test also confirmed that such a high level of phylogenetic congruence is quite unlikely to occur by chance (Figure S3).

### Strict Host-Symbiont Cocladogenesis in the Evolutionary Course of the Plataspid Stinkbugs

These results indicated that the evolutionary history of the gut symbiotic bacteria mirrors that of their host plataspid stinkbugs; the pattern so-called cospeciation or cocladogenesis. Similar cocladogenetic patterns have been reported from diverse insect taxa associated with obligate endocellular bacteria [8–15]. To our knowledge, this study is the first to identify a strict cocladogenesis between a group of insects and their gut symbionts. The phylogenetic congruence strongly suggests that a single bacterial infection in the common ancestor of the plataspid stinkbugs has been stably maintained over evolutionary time without horizontal transfers and has been diversified in parallel with the host speciation.

### Basis of the Host-Symbiont Cocladogenesis: Association by Descent or Association Maintained by Compatibility?

It is widely thought that the strict maternal transmission is the primary basis of host-symbiont cocladogenesis in insect endocellular symbionts [8–15]. In the plataspid stinkbugs, their symbionts are, although extracellularly located in the gut cavity, stably transmitted from mothers to offspring (see Table S1). The specialized traits for retainment and transmission of the symbiont, the isolated posterior midgut and the capsule production, are probably responsible for the host-symbiont coherence over evolutionary time. However, stable vertical transmission alone may be not sufficient to account for the strict host-symbiont cocladogenesis, since the ecology of the plataspid stinkbugs is potentially susceptible to accidental horizontal transfers of the symbiont. In the fields, several egg masses are frequently deposited on the same plant buds by different mothers of *M. punctatissima,* where intra-specific horizontal transfers of their symbionts can occur [22]. In Japan, M. cribraria and B. vahlii utilize the same host plant *Pueraria montana,* and M. punctatissima and Coptosoma semiflavum are found on the same host plant Pueraria lobata [35], which might provide opportunities for inter-specific horizontal transfers of their symbionts. In this context, genetic diversity of the symbiont in natural populations of the plataspid stinkbugs is of interest. Probably, strict maternal transmission is the primary basis of the host-symbiont cocladogenesis in the plataspid stinkbugs, but involvement of other factors, such as host-symbiont physiological compatibility, should also be taken into account.

### General Patterns in the Genome Evolution of Endocellular Symbiotic Bacteria

Recent molecular evolutionary analyses have suggested that the endocellular lifestyle of obligate insect symbionts has strongly affected their genome evolution, causing AT-biased nucleotide composition, accelerated rate of molecular evolution, and significant genome reduction [16,17]. These peculiar genetic traits are hypothesized to be the consequence of attenuated purifying selection due to small population size and strong bottleneck, which are associated with the lifestyle of vertically transmitted endocellular symbionts [17,18]. Here, it should be noted that small population size and strong bottleneck are also found in vertically transmitted extracellular symbionts like those of the plataspid stinkbugs. Thus, molecular evolutionary analyses of the plataspid symbionts will provide an opportunity to sort out the principal factor responsible for the reductive genome evolution. If the population genetic attributes such as small population size and bottleneck have the principal effect, the extracellular symbionts will also exhibit the peculiar genetic traits. If the endocellular environment itself is the principal factor, the peculiar genetic traits will be less conspicuous or absent in the extracellular symbionts.

### AT-Rich *16S rRNA* Genes of the Plataspid Symbionts

Table S2 shows the base composition of *16S rRNA* genes from representatives of the γ-*Proteobacteria.* Free-living bacteria exhibited AT contents around 45%, while obligate endocellular bacteria of various insects showed remarkably higher values, mostly over 50% and up to 64%. The extracellular symbionts of the plataspid stinkbugs exhibited values ranging from 50% to 54%, which were equivalent to those of the endocellular insect symbionts.

### Accelerated Molecular Evolution of the Plataspid Symbionts

Table 2 summarizes the results of relative rate tests for the *16S rRNA* gene sequences from the lineages of *Buchnera,* plataspid symbionts, and related free-living bacteria. The molecular evolutionary rate in the lineage of the extracellular symbionts was significantly higher (about 6.3-fold) than that of the free-living bacteria and was similar to that in the lineage of the aphid endocellular symbionts *Buchnera.*

**Table 2:**
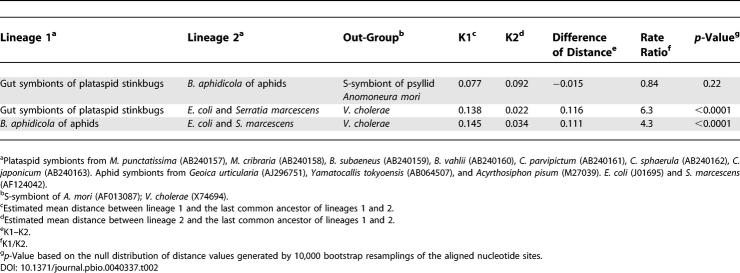
Relative Rate Tests for Comparing the Molecular Evolutionary Rate of *16S rRNA* Gene between the Lineages of Gut Symbionts of Plataspid Stinkbugs, Endocellular Symbionts of Aphids *(B. aphidicola),* and Their Free-Living Relatives

### Reduced Genome Size of the Plataspid Symbionts

**Figure 6:**
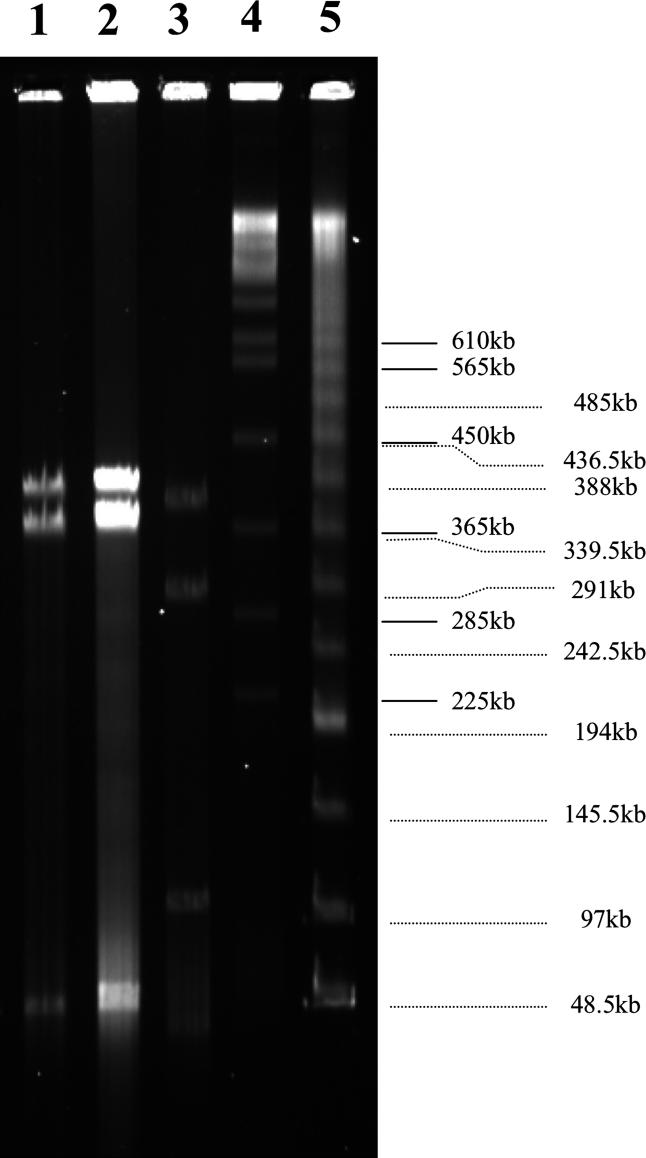
PFGE of the Symbiont Genomic DNA Prepared from the Posterior Midgut of a Female Adult of the Plataspid Stinkbugs Lane 1, *M. punctatissima;* lane 2, *M. cribraria;* lane 3, *C. parvipictum;* lane 4, yeast PFGE marker; lane 5, lambda PFGE marker. Marker sizes are shown on the right side.

Figure 6 shows the pulsed-field gel electrophoresis (PFGE) of the genomic DNA of the plataspid symbionts. The estimated genome sizes of the symbionts were 0.82 Mb, 0.82 Mb, and 0.83 Mb for *M. punctatissima, M. cribraria,* and *C. parvipictum,* respectively (Table S3). The genome sizes were significantly smaller than those of free-living γ-proteobacteria such as Escherichia coli (4.6 Mb) [36], Vibrio cholerae (4.0 Mb) [37], and Pseudomonas aeruginosa (6.3 Mb) [38] and were almost equivalent to those of endocellular symbionts such as *Buchnera* (0.45–0.65 Mb) [39], *Wigglesworthia* (0.70 Mb) [6], *Blochmannia* (0.81 Mb) [40], and *Baumannia* (0.69 Mb) [41].

### Genome Evolution of the Extracellular Symbiotic Bacteria of Plataspid Stinkbugs

These results demonstrated that the extracellular symbionts of the plataspid stinkbugs commonly exhibit the peculiar evolutionary patterns, AT-biased gene, accelerated molecular evolution, and reduced genome size, as has been reported from endocellular symbionts of diverse insects. This finding strongly favors the hypothesis that attenuated purifying selection due to small population size and strong bottleneck is the principal factor shaping the peculiar genetic traits of insect endosymbionts [17,18]. It should be noted that similar genetic features are found among obligate bacterial pathogens such as *Rickettsia, Chlamydia,* and *Mycoplasma,* and some of them are endocellular but not all [16,17]. Hence, the same evolutionary logic may apply not only to the microbial mutualists but also to the microbial parasites. Considering that the plataspid symbionts constitute a sister group of *Buchnera* (Figure 2), comparative genomic analyses of the extracellular and endocellular symbionts would provide further insights into how their symbiotic lifestyles have affected their molecular, evolutionary, and genomic features.

### Evolutionary Origin of Symbiont Capsule?

The evolutionary origin of the symbiont capsule is an enigma. The impressive similarities of the symbiotic organization between the different genera, *Megacopta, Brachyplatys,* and *Coptosoma* (Figure 1), strongly suggest that the capsule transmission system had already evolved in the common ancestor of these plataspid genera. No related stinkbug taxa have similar symbiont containers for their offspring. Therefore, the symbiont capsule must have evolved in the lineage of the family Plataspidae, in which about 530 species and 56 genera have been described thus far [42,43]. Wider survey of plataspid stinkbugs for their symbiotic system will shed light on the evolutionary origin of the unique mechanism for symbiont transmission.

### Proposal of Candidate Name

On account of the phylogenetically and biologically distinct traits as described above, we propose the designation “*Candidatus* Ishikawaella capsulata” for the symbiotic bacteria of the plataspid stinkbugs.

(i) Diagnostic features: The symbiont belongs to the γ-subclass of the *Proteobacteria* (Figure 2). The alignment of *16S rRNA* gene sequences from 12 isolates of the symbiont (representing three genera, seven species, and 12 populations of the host insects) plus other proteobacteria indicates distinctive residues, including the following (numbering based on the E. coli sequence): AUA at positions 455 to 457, A(A/G)AA at positions 830 to 833, UU(U/C)UU at positions 853 to 857, UUAU(A/G) at positions 1,006 to 1,010, and U(A/G)UAA at positions 1,019 to 1,023. The *16S rRNA* sequences reported here are AB240157–AB240163 and AB244765–AB244769. Also available are a *16S rRNA* sequence (AB067723) and a partial *groEL* sequence (AB231904) of the symbiont from M. punctatissima [22].

The symbiont lives in the cavity of posterior midgut of the host insects (Figure 1). Upon vertical transmission, the symbiont is packaged in symbiont capsule and deposited by female adult with eggs (Figure S1) [22, 26–28]. Electron microscopy shows that the symbiont is a globular-shaped bacterium, with cells 1–3 μm in diameter [28]. The size and shape of the symbiont vary depending on developmental stages of and locations in the host insects [26,28]. The symbiont genes exhibit high AT contents: 50.1% −54.1% for *16S rRNA* gene (Table S2) and 61.1% for *groEL* gene. The symbiont genome size is around 0.8 Mb (Figure 6; Table S3).

(ii) Hosts: The symbiont is associated with stinkbugs of the family Plataspidae, including *M. punctatissima, M. cribraria, B. subaeneus, B. vahlii, C. parvipictum, C. sphaerula,* and C. japonicum (Table S4). Probably the symbiont is also found in other plataspid stinkbugs including *C. scutellatum,* although no molecular data are available for them [26,27]. The host phylogeny is highly concordant with the symbiont phylogeny (Figure 5), suggesting stable host-symbiont association over evolutionary time. The symbiont is essential for normal growth and development of the host insects. Elimination of the symbiont results in abnormalities of the host insects, including mortality and sterility (Figures 3 and 4; Table 1) [22, 27]. The symbiont has not been cultured outside the host insects.

(iii) Nomenclature: The generic name honors Hajime Ishikawa, who pioneered molecular biological studies on insect symbiosis and passed away recently [44]. The specific name refers to the “capsule” encasing the symbiont.

### Plataspid Stinkbug as Model System for Symbiosis Studies

The unique symbiotic system of the plataspid stinkbugs, wherein host eggs and symbiont capsules are separable by using forceps under binocular microscope, enables novel experimental approaches to previously untouched aspects of the insect-microbe mutualism. For example, egg/capsule ratios in an egg mass can be freely altered, by which the levels of maternal investment in symbiont transmission can be experimentally manipulated. By exchanging eggs and capsules between populations, species, and genera of the insects, the extent of host-symbiont coevolution and coadaptation would be experimentally evaluated. Biochemical and nutritional analyses of isolated capsules would lead to understanding of the nature of symbiont inoculum upon vertical transmission. Isolated capsules can be the source of pure symbiont DNA needed for genome sequencing. We expect that, in addition to the aphid-*Buchnera* and tsetse-*Wigglesworthia* systems, the plataspid stinkbugs and their *Ishikawaella* symbionts provide an excellent model system for insect symbiosis studies.

## Materials and Methods

### Collection and rearing of insects.

Table S4 lists the plataspid stinkbugs used in this study, which represent three genera, seven species, and 12 populations. Adult insects collected in the fields were preserved in acetone for molecular analyses. Of these, four species, *M. punctatissima, M. cribraria, B. subaeneus,* and C. parvipictum were reared in the laboratory for experimental analyses. As for *M. punctatissima, M. cribraria,* and *B. subaeneus,* a pair of adult female and male was kept in a Petri dish (9 cm in diameter and 2 cm in depth) and fed with pea pods *(Pisum sativum).* As for *C. parvipictum,* a pair of adult female and male was reared on potted plants of buckwheat *(Fagopyrum esculentum).* They were maintained at 25 °C under a long day regimen (16L:8D). Egg masses produced by these insect pairs were used for experiments.

### Dissection of insects.

The adult insects were dissected by using forceps and scissors under a binocular microscope. The symbiotic organ, posterior midgut, was dissected out and immediately subjected to DNA extraction. From the egg masses, eggs and capsules were carefully isolated by using fine forceps under a binocular microscope.

### DNA extraction, cloning, genotyping, and sequencing.

The insect tissues, eggs, and capsules were subjected to DNA extraction by using the QIAamp DNA Mini Kit (Qiagen, Valencia, California, United States). From the samples, bacterial *16S rRNA* gene was amplified by PCR with the primers 16SA1 (5′-AGAGTTTGATCMTGGCTCAG-3′) and 16SB1 (5′-TACGGYTACCTTGTTACGACTT-3′). Insect mitochondrial *16S rRNA* gene was amplified with the primers mt16SA1 (5′-AAWAAACTAGGATTAGATACCCTA-3′) and mt16SB1 (5′-TCTTAATYCAACATCGAGGTCGCAA-3′). PCR was conducted with Ampli*Taq* DNA polymerase (Roche, Basel, Switzerland) and its supplemented buffer system under a temperature profile of 94 °C for 4 min followed by 30 cycles of 94 °C for 30 s, 55 °C for 1 min, and 72 °C for 2 min. The PCR products were cloned with pT7Blue T-vector (Novagen, Madison, Wisconsin, United States) and E. coli DH5α competent cells (Takara Bio, Shiga, Japan). The plasmid inserts were amplified by PCR with the primers Univ19 (5′-GTTTTCCCAGTCACGACGT-3′) and Rev20 (5′-AGCTATGACCATGATTACGC-3′) for checking the product size (approximately 1.6 kb for both of the genes). The amplified inserts of the bacterial *16S rRNA* gene were subjected to restriction fragment length polymorphism genotyping by using restriction endonucleases *HaeIII* and *RsaI*. The inserted plasmids were purified by using the QIAprep Spin Miniprep Kit (Qiagen) and were subjected to DNA sequencing as described [45].

### Molecular phylogenetic analysis.

Multiple alignments of the DNA sequences were generated by using the program Clustal W [46]. The alignments were then inspected and corrected manually, from which ambiguously aligned sites were removed. Phylogenetic analyses were conducted by the three methods, MP, ML, and Bayesian. MP and ML trees were constructed by using the program PAUP 4.0b10 [47]. In the MP analysis, all sites and character changes were weighted equally. In the ML analysis, we selected the GTR + I + G model for the symbiont phylogeny and TVM + G model for the host phylogeny, on the basis of the Akaike criterion estimated by the program Modeltest 3.06 [48]. Bootstrap tests were performed with 100 replications in the MP and ML analyses. In the Bayesian analysis, we used the programs MrBayes 3.0b4 [49] and MrModeltest v2.1 [50]. The Akaike criterion selected the GTR + I + G model for the symbiont phylogeny and the GTR + G model for the host phylogeny. In total, 9,000 trees were obtained (ngen = 90,000, samplefreq = 10), and the first 3,500 of these were considered as the “burn in” and discarded. Based on the data, a 50% majority-rule consensus tree was produced.

### Coevolutionary analysis.

The extent of phylogenetic congruence between the host and symbiont phylogenies was evaluated by the jungles algorithm equipped with the program TreeMap v2.02β [34] under the default setting of the event costs (codivergence = 0; duplication = host switch = sorting event = 1). Based on the host and symbiont Bayesian trees, the maximum number of codivergence events (the number of shared nodes between the trees) was parsimoniously estimated. Then, the statistical significance was evaluated by testing the null hypothesis that the observed number of codivergence events was not larger than the expected number of codivergence events between the observed host tree and 1,000 randomly generated trees.

### Relative rate test.

Genetic distances were estimated under the HKY substitution model and the gamma distribution of rate variation across nucleotide sites (with eight categories) by using the program TREE-PUZZLE5.2 [51]. To take into account the phylogenetic relationship among the samples, the genetic distances between the lineages were calculated as weighted average [52]. The significance of the rate difference was evaluated by generating 10,000 bootstrap replicates of the alignment sites [53]. The rate differences between the lineages were estimated with each replicate data as described above. For each of the tests, we excluded all aligned sites that contained a gap or an unresolved base in any of the sequences considered.

### Experimental manipulation of egg masses.

Each of the egg masses deposited by the reared pairs of the insects was divided into two, each of which contained eggs and capsules of similar numbers, respectively. For example, an egg mass of M. punctatissima contains 22 eggs and six capsules on average (unpublished data). One of the halves was left untreated (control egg mass with capsules), while the other of the halves was deprived of all capsules by using forceps and a needle (treated egg mass without capsules). Each of the experimental egg masses was glued on a piece of filter paper and placed in a Petri dish (5 cm in diameter and 1 cm in depth) and humidified with a wet cotton ball at room temperature (23–26 °C) until hatch.

### Confirmation of symbiont elimination by specific PCR detection.

1 d after hatching of the experimental egg masses, newborn nymphs were collected and subjected to DNA extraction. The following primer sets were used for specific detection of the stinkbug symbionts: MRKM16SF (5′-TATAGAGATATATAAGTGCCTTTCG-3′) and 16SB1 for *M. punctatissima, M. cribraria,* and *C. parvipictum;* and 16SA1 and TYMR16SR (5′-CCCTTCATGAAACTCTAGCCTATT-3′) for *B. subaeneus.* The PCR temperature profile was 94 °C for 4 min followed by 30 or 35 cycles of 94 °C for 30 s, 55 °C for 1 min, and 72 °C for 2 min.

### Fitness measurement.

From each of the experimental egg masses with and without capsules, newborn nymphs were picked up and subjected to fitness measurements. As for M. punctatissima and *M. cribraria,* ten nymphs were randomly chosen from each of the experimental egg masses and reared on a potted soybean plant *(Glycine max).* As for B. subaeneus and *C. parvipictum,* all nymphs (5–16 insects) from each of the experimental egg masses were reared on pea pods and buckwheat plants, respectively. Survival rate and developmental stage of the nymphs were recorded every 10 d until all the nymphs either became adults or died. Thorax width of the emerged adults was measured by using a digital caliper (NSK, 950–101 Max-15) to the nearest 0.01 mm under a binocular microscope.

### PFGE.

Agarose plugs containing the symbiont cells were prepared by mixing homogenized posterior midgut from a female insect with 1% low melting point agarose. After an overnight treatment with proteinase K, the agarose-embedded DNA was digested with restriction enzyme *ApaI.* PFGE was conducted by using Genofield AE-8900 (ATTO) according to the manufacturer's instruction.

## Supporting Information

Figure S1 Upper View (Left) and Lower View (Right) of an Egg Mass of Plataspid Stinkbugs (A and B) *M. cribraria;* (C and D) *B. subaeneus;* (E and F) *C. parvipictum.* Bars, 1 mm. Symbiont capsules (arrowheads) are seen from the lower side.
(268 KB PDF)

Figure S2 Diagnostic PCR Detection of Symbiont Acquisition by Newborn Nymphs of C. parvipictum Hatched from the Experimental Egg Masses with and without Capsules (A) Mitochondrial *16S rRNA* gene of the host insect; (B) bacterial *16S rRNA* gene of the symbiont. Lanes 1–6, individual nymphs from a control egg mass portion with capsules; lanes 7–11, individual nymphs from a treated egg mass portion without capsule; lane 12, no template control; lane 13, an egg before hatch; lane 14, symbiont capsules; lane M, DNA size markers (1.5, 1.0, 0.9, 0.8, 0.7, 0.6, 0.5, 0.4, and 0.3 kb from top to bottom).
(96 KB PDF)

Figure S3 Randomization Test for the Phylogenetic Congruence between the Plataspid Stinkbugs and Their Gut Symbionts Frequency distribution of the number of cospeciation events between the observed host tree and each of 1,000 randomly generated trees is shown. Arrow indicates the observed number of cospeciation events (see Figure 5).
(84 KB PDF)

Table S1 Acquisition of the Symbiotic Bacterium by Newborn Nymphs from Experimental Egg Masses with and without Capsules (68 KB DOC)

Table S2 AT Contents of *16S rRNA* Gene of the Gut Symbiotic Bacteria of the Plataspid Stinkbugs, the Obligate Primary Endocellular Symbiotic Bacteria of Various Insects, and Free-Living Bacteria Representing the γ-Subclass of the *Proteobacteria*
(77 KB PDF)

Table S3 DNA Fragments Detected by PFGE of the Plataspid Symbiont Genomes (37 KB DOC)

Table S4 Plataspid Stinkbugs Used in This Study (63 KB DOC)

Video S1 A Newborn Nymph of M. punctatissima Looking for Symbiont Capsule An experimentally constructed egg mass on a filter paper, which consists of three eggshells and a capsule. A newborn nymph is looking for symbiont capsule, with its proboscis stretching forward. When locating a capsule, the nymph probed the capsule with its proboscis, agitating its own body vigorously. Finally, the nymph started ingesting the symbiont, with its antennae oriented laterally.
(9.6 MB MOV)

### Accession Numbers

The DNA sequences reported in this paper have been deposited in the DNA Data Bank of Japan database (http://www.ddbj.nig.ac.jp) accession numbers AB240157–AB240170, AB240572–AB240574, AB240576, and AB244765–AB244769. The accession numbers for some of the species discussed in this paper are: plataspid stinkbugs B. subaeneus (AB240166), B. vahlii (AB240167), C. japonicum (AB240170), C. parvipictum (AB240168), C. sphaerula (AB240169), M. cribraria (AB240165), and M. punctatissima (AB240164); other stinkbugs Agonoscelis nubila (AB240572), Paromius exiguus (AB240576), Plautia crossota (AB240573), and Poecilocoris lewisi (AB240574); primary symbionts Buchnera aphidicola from aphids Acyrthosiphon pisum (M27039), Geoica urticularia (AJ296751), and Yamatocallis tokyoenis (AB064507); gut symbionts Ishikawaella capsulata from plataspid stinkbugs B. subaeneus (AB240159), B. vahlii (AB240160, AB244767), C. japonicum (AB240163), C. parvipictum (AB240161, AB244768, AB244769), C. sphaerula (AB240162), *M. cribraria* (AB240158), and M. punctatissima (AB240157, AB244765, AB244766); other insect symbionts Arsenophonus arthropodicus from louse fly Pseudolynchia canariensis (DQ115536), Baumannia cicadellinicola from sharpshooter Homalodisca coagulata (AF465793), Blochmannia floridanus from ant Camponotus floridanus, Carsonella rudii from psyllid Anomoneura mori (AB013086), Hamiltonella defensa from aphid A. pisum (AF293616), Nardonella sp. from weevil Metamasius hemipterus (AY126635), Portiera aleyrodidarum from whitefly Bemisia tabaci (Z11925), Regiella insecticola from aphid A. pisum (AF293618), Serratia symbiotica from aphid A. pisum (M27040), Sodalis glossinidius from tsetse fly Glossina morsitans (AP008232), Wigglesworthia glossinidia from tsetse fly Glossina brevipalpis (AB063521), primary symbiont of weevil Sitophilus oryzae (AF005235), secondary symbiont of psyllid Anomoneura mori (AF013087), and secondary symbiont of mealybug Antonina crawii (AB030020); other bacteria E. coli (J01695), Neisseria gonorrhoeae (X07714), Photorhabdus luminescens (AJ007404), Pseudomonas aeruginosa (AB037545), Salmonella typhi (U88545), S. marcescens (AF124042); V. cholerae (X74694), Yersinia pestis (AE013602), and Zymobacter palmae (D14555).

## Abbreviations

ML: maximum likelihood
MP: maximum parsimony
PFGE: pulsed-field gel electrophoresis
TCM: thin crypt-bearing midgut section
