# Supplementary material for: Strict Host-Symbiont Cospeciation and Reductive Genome Evolution in Insect Gut Bacteria
Source: PLoS Biol. 2006 Oct 10;4(10):e337. doi: 10.1371/journal.pbio.0040337 (PMC1592312; doi:10.1371/journal.pbio.0040337)
Supplement: Supplementary file 1 [file pbio.0040337.sg001.pdf]

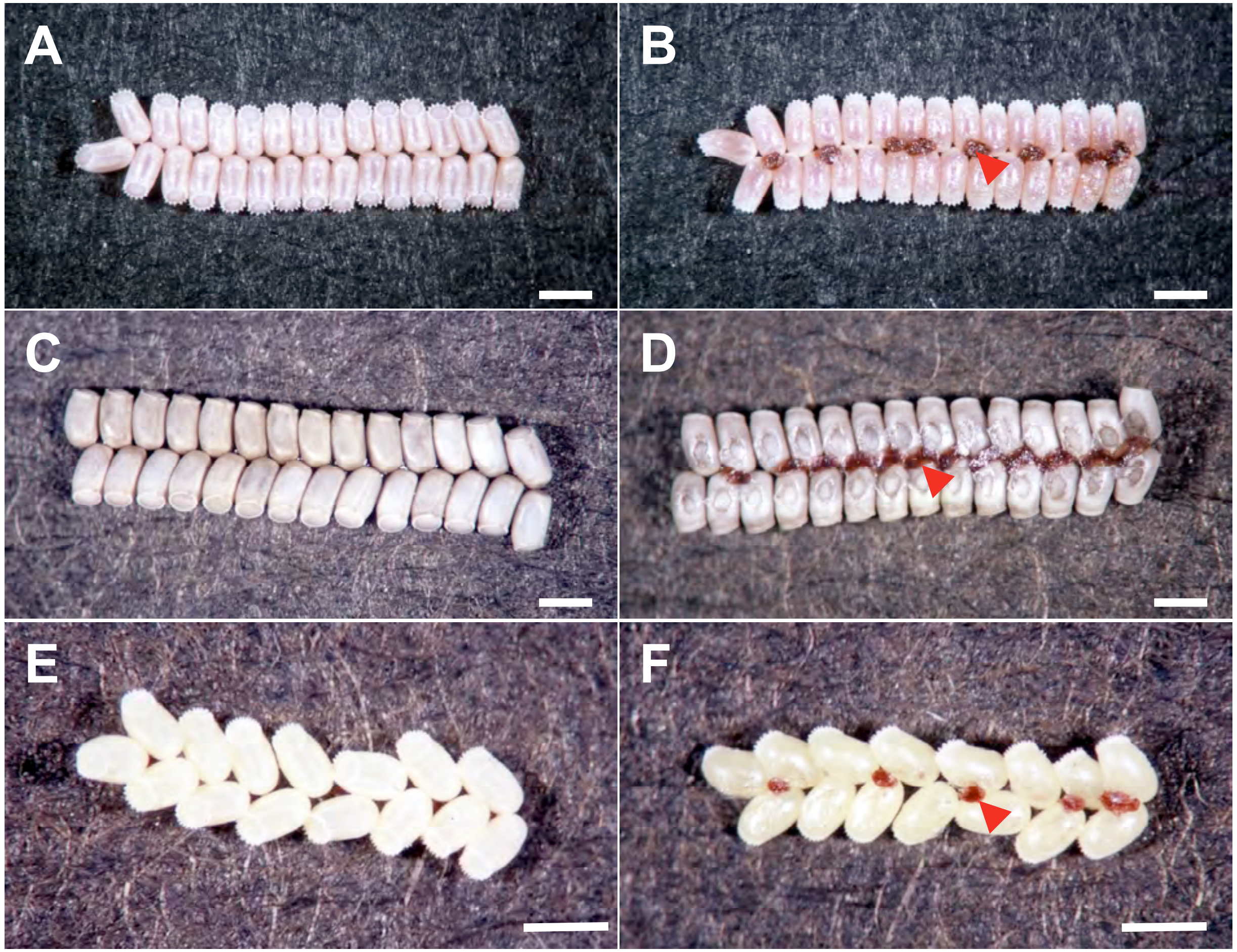

Fig. S1. Upper view (left) and lower view (right) of an egg mass of plataspid stinkbugs.
(A) and (B), *M. cribraria*;
(C) and (D), *B. subaeneus*;
(E) and (F), *C. parvipictum*.
Bars,1mm.
Symbiont capsules (arrowheads) are seenfrom the lower side.
