# Supplementary material for: Strict Host-Symbiont Cospeciation and Reductive Genome Evolution in Insect Gut Bacteria
Source: PLoS Biol. 2006 Oct 10;4(10):e337. doi: 10.1371/journal.pbio.0040337 (PMC1592312; doi:10.1371/journal.pbio.0040337)
Supplement: Supplementary file 2 [file pbio.0040337.sg002.pdf]

(A)

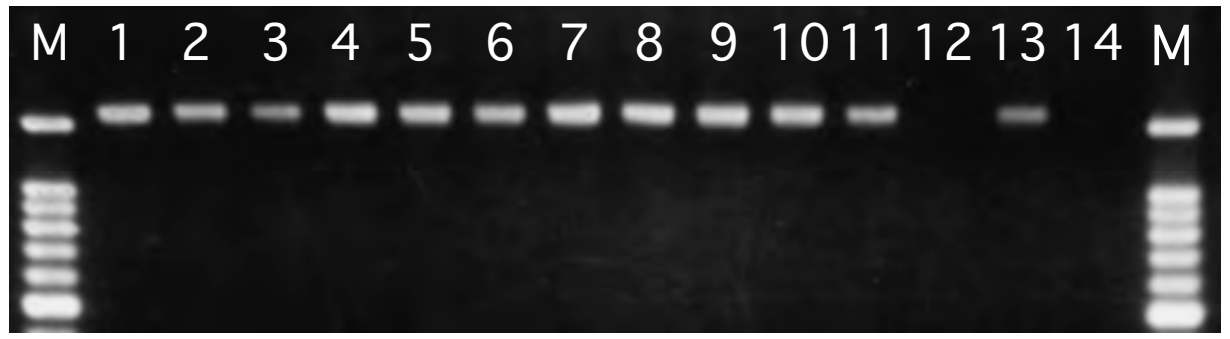

(B)

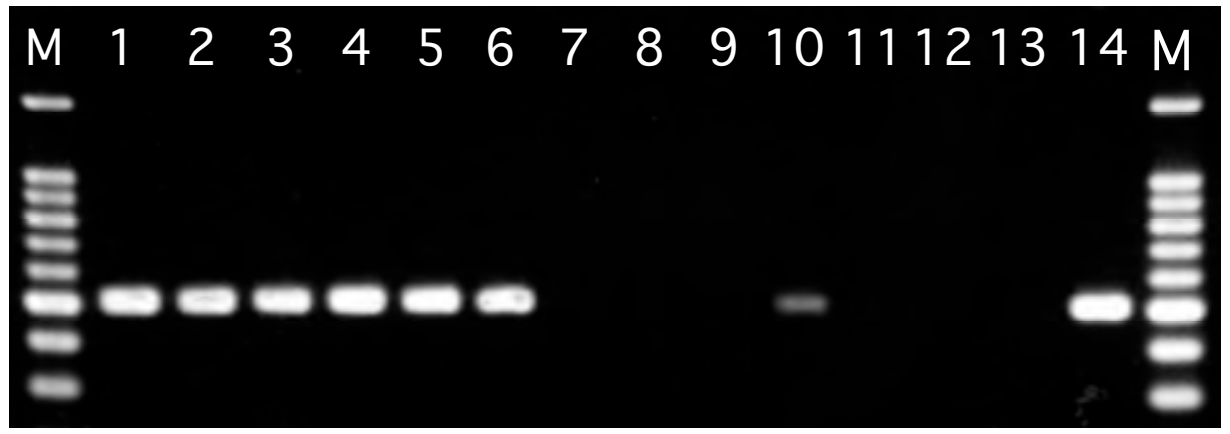

Fig. S2. Diagnostic PCR detection of symbiont acquisition by newborn nymphs of *C. parvipictum* hatched from the experimental egg masses with and without capsules. (A) Mitochondrial *16S rRNA* gene of the host insect; (B) bacterial *16S rRNA* gene of the symbiont. Lanes 1-6, individual nymphs from a control egg mass portion with capsules; lanes 7-11, individual nymphs from a treated egg mass portion without capsule; lane 12, no template control; lane 13, an egg before hatch; lane 14, symbiont capsules; lane M, DNA size markers (1.5, 1.0, 0.9, 0.8, 0.7, 0.6, 0.5, 0.4, and 0.3 kb from top to bottom).
