# Supplementary material for: Strict Host-Symbiont Cospeciation and Reductive Genome Evolution in Insect Gut Bacteria
Source: PLoS Biol. 2006 Oct 10;4(10):e337. doi: 10.1371/journal.pbio.0040337 (PMC1592312; doi:10.1371/journal.pbio.0040337)
Supplement: Supplementary file 3 [file pbio.0040337.sg003.pdf]

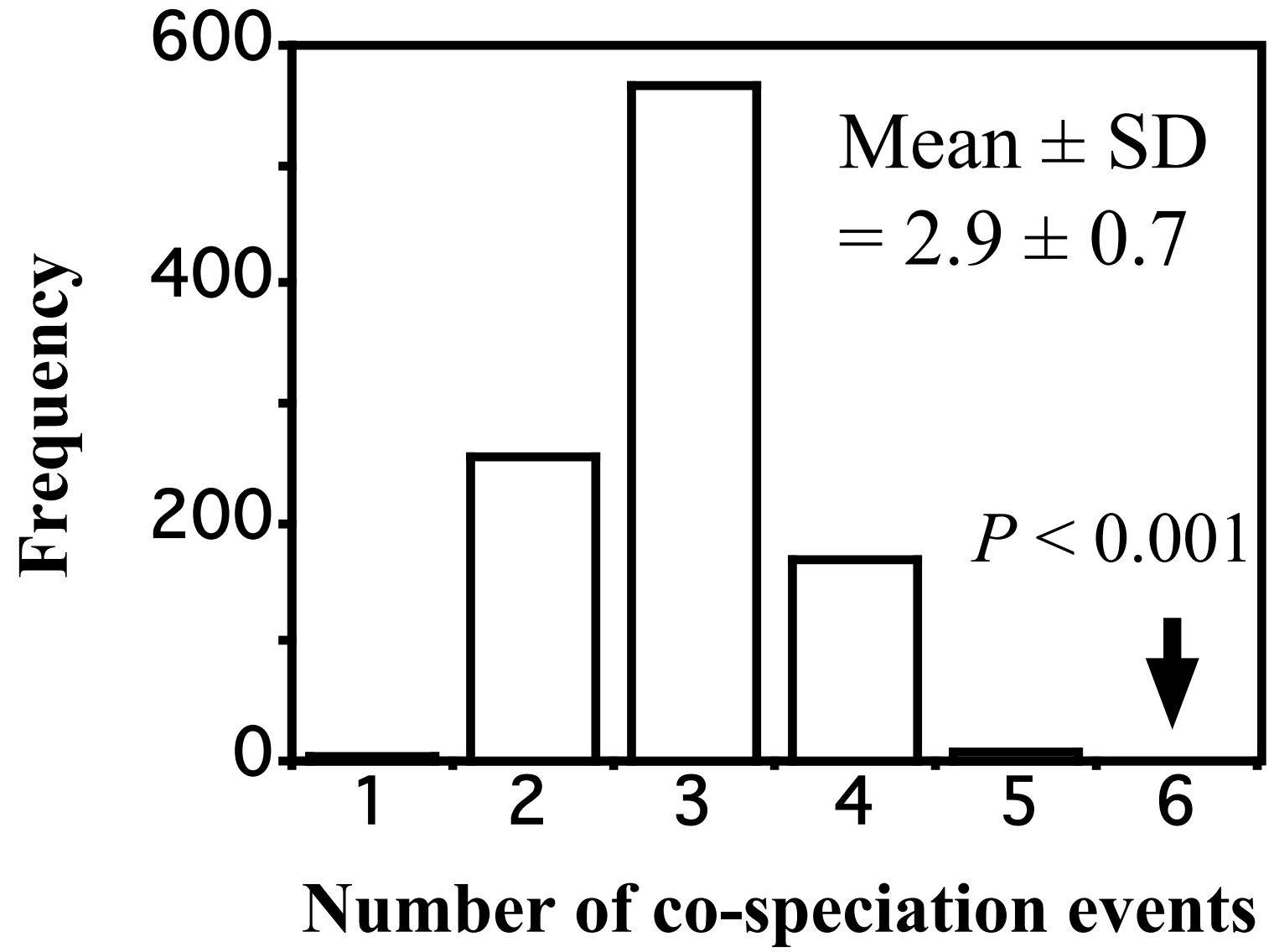

Fig. S3. Randomization test for the phylogenetic congruence between the plataspid stinkbugs and their gut symbionts. Frequency distribution of the number of co-speciation events between the observed host tree and each of 1,000 randomly-generated trees is shown. Arrow indicates the observed number of co-speciation events (see Figure 5).
