# Supplementary material for: Strict Host-Symbiont Cospeciation and Reductive Genome Evolution in Insect Gut Bacteria
Source: PLoS Biol. 2006 Oct 10;4(10):e337. doi: 10.1371/journal.pbio.0040337 (PMC1592312; doi:10.1371/journal.pbio.0040337)
Supplement: Supplementary file 4 [file pbio.0040337.st002.pdf]

Table S2. AT contents of *16S rRNA* gene of the gut symbiotic bacteria of the plataspid stinkbugs, the obligate primary endocellular symbiotic bacteria of various insects, and free-living bacteria representing the γ-subclass of the *Proteobacteria*.

SYMBIONT	HOST INSECT	AT CONTENT (%)
**Obligate gut symbiont of plataspid stinkbugs**		
Ishikawaella capsulata	*Brachyplatys subaeneus*	53.9
Ishikawaella capsulata	*Brachyplatys vahlii*	54.1
Ishikawaella capsulata	*Coptosoma japonicum*	51.9
Ishikawaella capsulata	*Coptosoma parvipictum*	50.1
Ishikawaella capsulata	*Coptosoma sphaerula*	51.9
Ishikawaella capsulata	*Megacopta cribraria*	50.8
Ishikawaella capsulata	*Megacopta punctatissima*	50.7
**Obligate endocellular symbiont of various insects**		
Buchnera aphidicola	*Acyrthosiphon pisum* (aphid)	49.8
Buchnera aphidicola	*Geoica urticularia* (aphid)	51.3
Buchnera aphidicola	*Yamatocallis tokyoensis* (aphid)	52.1
Baumannia cicadellinicola	*Homalodisca coagulata* (sharpshooter)	51.3
Blochmannia floridanus	*Camponotus floridanus* (ant)	51.9
Carsonella ruddii	*Anomoneura mori* (psyllid)	64.0
Nardonella sp.	*Metamasius hemipterus* (weevil)	60.4
Portiera aleyrodidarum	*Bemisia tabaci* (whitefly)	56.0
Wigglesworthia glossinidia	*Glossina morsitans* (tsetse fly)	51.5
**Free living bacteria**		
Enterobacter aerogenes[a]		45.1
Erwinia carotovora[b]		45.8
Escherichia coli		45.6
Pseudomonas aeruginosa		45.5
Salmonella typhi		45.6
Serratia marcescens		45.0
Vibrio cholerae		45.9

Accession numbers not indicated in Fig. 2: [a]AB004750; [b]Z96089.
