# Supplementary material for: Strict Host-Symbiont Cospeciation and Reductive Genome Evolution in Insect Gut Bacteria
Source: PLoS Biol. 2006 Oct 10;4(10):e337. doi: 10.1371/journal.pbio.0040337 (PMC1592312; doi:10.1371/journal.pbio.0040337)
Supplement: Supplementary file 5 [file pbio.0040337.st001.doc]

Table S1. Acquisition of the symbiotic bacterium by newborn nymphs from experimental egg masses with and without capsules.

| Egg mass  number | *M. punctatissima* | |  | *M. cribraria* | |  | *B. subaeneus* | |  | *C. parvipictum* | |
| --- | --- | --- | --- | --- | --- | --- | --- | --- | --- | --- | --- |
|  | With capsule^*1^ | No capsule^*2^ |  | With capsule | No capsule |  | With capsule | No capsule |  | With capsule | No capsule |
| 1 | 10/10^*3^ | 0/10 |  | 10/10 | 0/10 |  | 10/10 | 1^*4^/9 |  | 10/10 | 0/9 |
| 2 | 9/10 | 0/10 |  | 10/10 | 0/10 |  | 12/12 | 0/14 |  | 7/7 | 0/4 |
| 3 | 10/10 | 3^*4^/10 |  | 10/10 | 0/10 |  | 17/17 | 1^*4^/16 |  | 6/6 | 0/6 |
| 4 | 10/10 | 0/10 |  | 10/10 | 0/10 |  | 12/12 | 2^*4^/11 |  | 6/6^*5^ | 1^*4^/5^*5^ |
| 5 | 10/10 | 1^*4^/10 |  | 10/10 | 3^*4^/10 |  | 12/12 | 8^*4^/12 |  | 7/7 | 0/6 |
| 6 | - | - |  | - | - |  | 10/10 | 3^*4^/11 |  | 5/5 | 0/5 |
| Total | 49/50 | 4^*4^/50 |  | 50/50 | 3^*4^/50 |  | 73/73 | 15^*4^/73 |  | 41/41 | 1^*4^/35 |

^*1^ Experimental egg mass portion from which no symbiont capsule was removed.

^*2^ Experimental egg mass portion from which all symbiont capsules were removed.

^*3^ For example, 10/10 means 10 symbiotic nymphs per 10 nymphs examined by diagnostic PCR.

^*4^ All samples showed a faint band after 35 cycles of PCR.

^*5^ Results after 35cycles of PCR are shown in Figure S2.
