# Supplementary material for: Strict Host-Symbiont Cospeciation and Reductive Genome Evolution in Insect Gut Bacteria
Source: PLoS Biol. 2006 Oct 10;4(10):e337. doi: 10.1371/journal.pbio.0040337 (PMC1592312; doi:10.1371/journal.pbio.0040337)
Supplement: Supplementary file 6 [file pbio.0040337.st003.doc]

Table S3. DNA fragments detected by pulsed-field gel electrophoresis of the plataspid symbiont genomes.

Host plataspid	*Megacopta	Megacopta	Coptosoma*

stinkbug	*punctatissima	cribraria	parvipictum*

Size of	400 kb	400 kb	380 kb

fragments	365 kb	365 kb	300 kb

50 kb	50 kb	110 kb

35 kb

Total size	815 kb	815 kb	825 kb
