# Supplementary material for: Strict Host-Symbiont Cospeciation and Reductive Genome Evolution in Insect Gut Bacteria
Source: PLoS Biol. 2006 Oct 10;4(10):e337. doi: 10.1371/journal.pbio.0040337 (PMC1592312; doi:10.1371/journal.pbio.0040337)
Supplement: Supplementary file 7 [file pbio.0040337.st004.doc]

Table S4. Plataspid stinkbugs used in this study.

| Species | Collection site^*1^ | Host plant species (family) | Collection date | Collector | Nucleotide sequence accession number^*2^ |
| --- | --- | --- | --- | --- | --- |
| *M. punctatissima* | Tsukuba, Ibaraki | *Pueraria lobata* (Leguminosae) | July, 2003 | T. Hosokawa | AB240157 / AB240164 |
|  | Onomichi, Hiroshima | *Pueraria lobata* (Leguminosae) | May, 2001 | T. Fukatsu | AB244765 / --- |
|  | Yakushima Island, Kagoshima | *Pueraria lobata* (Leguminosae) | May, 2001 | N. Ijichi | AB244766 / --- |
| *M. cribraria* | Ishigaki, Okinawa | *Pueraria montana* (Leguminosae) | November, 2003 | F. Kuchiki | AB240158 / AB240165 |
| *B. subaeneus* | Ishigaki, Okinawa | *Canavalia cathartica* (Leguminosae) | June, 2003 | K. Kohno | AB240159 / AB240166 |
| *B. vahlii* | Onna, Okinawa | *Pueraria montana* (Leguminosae) | June, 2002 | T. Fukatsu | AB240160 / AB240167 |
|  | Ishigaki, Okinawa | *Canavalia cathartica* (Leguminosae) | June, 2003 | K. Kohno | AB244767 / --- |
| *C. parvipictum* | Saga, Saga | *Polygonum thunbergii* (Polygonaceae) | May, 2004 | M. Hironaka | AB240161 / AB240168 |
|  | Sakado, Saitama | No data | June, 2003 | Y. Kamimura | AB244769 / --- |
|  | Sukumo, Kohchi | No data | June, 2003 | M. Takai | AB244768 / --- |
| *C. sphaerula* | Ishigaki, Okinawa | unidentified species (Leguminosae) | July, 2002 | K. Kohno | AB240162 / AB240169 |
| *C. japonicum* | Tosa-Shimizu, Kohchi | *Lespedeza cuneata* (Leguminosae) | May, 2003 | M. Takai | AB240163 / AB240170 |

^*1^ All are in Japan (city, prefecture).

^*2^ Symbiont *16S rRNA* gene / Host mitochondrial *16S rRNA* gene.
